# Near Infrared Investigation of Polypropylene–Clay Nanocomposites for Further Quality Control Purposes—Opportunities and Limitations

**DOI:** 10.3390/ma8095272

**Published:** 2015-08-31

**Authors:** Andreas Witschnigg, Stephan Laske, Clemens Holzer, Raj Patel, Atif Khan, Hadj Benkreira, Phil Coates

**Affiliations:** 1 Department of Polymer Engineering and Science, Chair of Polymer Processing, Montanuniversitaet Leoben, Otto Gloeckel Strasse 2, Leoben 8700, Austria; E-Mails: Andreas.Witschnigg@tugraz.at (A.W.); Clemens.Holzer@unileoben.ac.at (C.H.); 2 School of Engineering, Design and Technology, University of Bradford, Richmond Road, Bradford BD7 1DP, UK; E-Mails: R.Patel@Bradford.ac.uk (R.P.); ahkahn@hotmail.com (A.K.); H.Benkreira@Bradford.ac.uk (H.B.); P.D.Coates@Bradford.ac.uk (P.C.)

**Keywords:** NIR spectroscopy, quality control, polypropylene nanocomposites

## Abstract

Polymer nanocomposites are usually characterized using various methods, such as small angle X-ray diffraction (XRD) or transmission electron microscopy, to gain insights into the morphology of the material. The disadvantages of these common characterization methods are that they are expensive and time consuming in terms of sample preparation and testing. In this work, near infrared spectroscopy (NIR) spectroscopy is used to characterize nanocomposites produced using a unique twin-screw mini-mixer, which is able to replicate, at ~25 g scale, the same mixing quality as in larger scale twin screw extruders. We correlated the results of X-ray diffraction, transmission electron microscopy, *G*′ and *G*″ from rotational rheology, Young’s modulus, and tensile strength with those of NIR spectroscopy. Our work has demonstrated that NIR-technology is suitable for quantitative characterization of such properties. Furthermore, the results are very promising regarding the fact that the NIR probe can be installed in a nanocomposite-processing twin screw extruder to measure inline and in real time, and could be used to help optimize the compounding process for increased quality, consistency, and enhanced product properties.

## 1. Introduction

Layered silicates, such as nanoclays, are the most common nanofiller used in the polymer processing industry due to their potential of enhancing material properties and the fact that they are less expensive when compared to other nanofillers like carbon nanotubes or fullerene. Additionally, due to fact that the nanoclays form of delivery are agglomerated tactoids in the range of microns before processing, the health risk is very low. The primary reason for the improvement of material property is the reinforcement of the polymer matrix by means of the particles. The layered inorganic filler platelets also have a restrictive effect on the polymer chains movability. Layered silicates (most common montmorillonite) with an aspect ratio up to 1000 are used to enhance Young’s modulus, tensile strength, barrier, and other mechanical or physical properties [1,2].

Various measurements are used for determining the formed structures (assessing intercalation *vs.* exfoliation) and the homogeneity of the filler, including optical microscopy, scanning (SEM) and transmission (TEM) electron microscopy, mechanical (tensile strength, extensional rheology), and scattering methods (small angle (SAXS) and wide angle (WAXS) X-ray scattering). Nevertheless, these are all time consuming, cost intensive, off-line methods and they do not have the possibility of being installed as an in-line measurement and quality control device directly in the production process. 

In this study, a more practical way to determine material homogeneity and properties was used by applying near infrared spectroscopy (NIR). NIR spectroscopy is an optical, non-destructive method used to obtain information about the composition of samples and interactions within the sample. The basic functionality of the NIR technology can be described as follows: a light source is used to transmit near infrared radiation through a sample. This radiation excites and vibrates the molecular bonds, resulting in an energy absorbance at specific wave lengths depending on the type of molecule and molecular bonding, which can be detected by NIR spectroscopy. The wavelength of the absorbance bands in the NIR spectrum provides the information for identification of substances and chemical functionalities. 

NIR measurements have a variety of successful applications in polymer science, including nanocomposites and the determination of the achievable properties, such as the analysis of polymerization or copolymerization (mostly done by detecting the characteristic absorption caused by chemical groups, such as OH groups or vinyl acetate groups in ethylene vinyl acetate), crystallinity, molecular weight, anisotropy, intermolecular interactions, molar mass, porosity, specific surface area, tacticity, orientation, concentrations of flame retardants (e.g., melamine cyanurate), density measurements, and other chemical processes that appear during polymer processing [3,4,5,6,7,8,9,10,11].

Regarding the evaluation of nanocomposites utilizing NIR, the prevailing conditions in the sample (chemical, physical bondings, and resulting structures) are closely linked with the properties, which can therefore be determined with the use of NIR spectroscopy [12,13,14,15]. Shinzawa *et al.* [16,17] examined the crystalline structure and mechanical properties, as well as the influence of UV-radiation on polylactic acid (PLA) clay nanocomposites by NIR spectroscopy. Barbas *et al.* [18] determined the degree of dispersion of polypropylene-clay nanocomposites utilizing an in-line NIR setup. Laske and Witschnigg *et al.* reported that in-line [19], as well as off-line [20], NIR is very suitable for detecting mechanical and rheological properties. Furthermore the analysis of the melt strength of a polypropylene (PP) nanocomposite with off-line NIR spectroscopy (correlated with off-line rheotens measurement) has also been achieved [20].

Following a more holistic approach, the scope of this paper is to apply NIR on a big number of off-line samples to ensure statistical stability with a large variety of properties and used characterization methods to show the possibilities and even more important the limits of this technology and gain information on possible one-for-all chemometric modeling strategies for quality control purposes.

## 2. Experimental Section

### 2.1. Materials

Two polypropylene types PP 575P (Sabic, Sittard, The Netherlands) with a MFI of 10.5 g/10 min (230 °C/2.16 kg) and Moplen HP420 (Lyondell Basell Industries, Rotterdam, The Netherlands) with a MFI of 8 g/10 min (230 °C/2.16 kg) were used. The processed nanofiller (organomodified montmorillonite clay) with commercial indication Cloisite 20A was supplied by Southern Clay Products Inc. (Gonzales, TX, USA). To achieve good interactions between the nonpolar PP and the polar nanofiller, a maleic anhydride compatibilizer Polybond 3200 (Crompton, Middlebury, CT, USA) referred to as PP-g-MA with a MFI of 115 g/10 min (190 °C/2.16 kg) and a maleic anhydride level of 1 wt. % was used. The nanofiller content was varied from 0 to 6 wt. % [21].

### 2.2. Production of PP-Nanocomposites 

The compounding experiments were done on the twin-screw mini-mixer developed at the University of Bradford [14]. This device was built in order to achieve high levels of shear and elongation to break up the organoclay particles into dispersed stacks of silicate tactoids or even thin stacks of layered silicates for very small samples (~25 g) [22]. A ¼ fractional factorial experiment that provides sufficient degrees of freedom to resolve all main parameter effects as well as all two-factor interaction was utilised. The experimental design of experiments (DoE) was generated using Design-Expert 7.0 (Stat-Ease Inc., Minneapolis, MN, USA). Table 1 describes the DoE runs [21].

**Table 1:** The DoE run order of the experiments [21].

Run	Speed (rpm)	Residence Time (min)	Temperature (°C)	Nanoclay Loading (%)	Compatibiliser Loading (%)	MFR (g/10 min)
1	60	8	190	6	2	8
2	20	2	190	6	2	10.5
3	60	8	230	2	6	8
4	60	2	190	6	6	10.5
5	20	8	190	2	6	10.5
6	20	8	190	6	6	8
7	60	8	230	6	6	10.5
8	20	2	230	2	6	10.5
9	20	2	230	6	6	8
10	20	8	230	2	2	8
11	40	5	210	4	4	8
12	20	8	230	6	2	10.5
13	60	8	190	2	2	10.5
14	60	2	230	6	2	8
15	20	2	190	2	2	8
16	60	2	190	2	6	8
17	40	5	210	4	4	10.5
18	60	2	230	2	2	10.5
19	20	2	190	0	2	8
20	40	5	210	0	4	8
21	60	8	230	0	6	10.5

The polymer nanocomposite output from each DoE was used to investigate the mechanical properties, morphology, and rheology [21], as well as the NIR studies reported here.

### 2.3. Production of Test Specimen

The near infrared spectroscopy measurements were performed on samples from the DoE runs using plates with a thickness of 2 mm. For the production of these plates a hydraulic vacuum press machine (Collin 200 PV, Dr. Collin, Ebersberg, Germany) was used.

### 2.4. Mechanical Properties

Tensile testing was performed according to BS EN ISO 527-1: 1996 using an Instron 5564 Universal Tester (Instron, Buckinghamshire, UK) with a clip-on extensometer at a cross-head speed of 50 mm/min. At least five tests were completed for each run.

### 2.5. Structural Analysis

A Philips X’Pert type PW3040 was used to determine the D-spacing between clay platelets in the materials. The X-ray beam was Cu K_α1_ (λ = 1.5418 Å), and data were collected from 1° to 40° [21]. 

For the transmission electron microscopy (TEM), nanotomes of 50–70 nm thickness were cut and investigated using a Philips CM100 TEM operated at an accelerating voltage of 100 kV. The cutting of the nanotomes was performed on a strip with 0.5 mm × 0.5 mm × 5 mm of the nanocomposite embedded in a cured epoxy resin using a DiATOME diamond knife. One sample was analysed for each DoE Run [21].

### 2.6. Rheological Investigations

To gain insight into the shear and extensional flow behaviour of the various compounds, rheological investigations have been undertaken to assess the effect of the dispersed nanoparticles on the flow. The analysis was performed on the GeminiTM 200 Advanced Rheometer (Malvern Instruments Ltd., Malvern, UK) operated with parallel plate geometry at a shear rate of 0.01 to 100 s^−1^ [21].

### 2.7. Fourier Transform Near Infrared (FT-NIR) Measurements

For NIR measurements a Fourier transform near infrared (FT-NIR) spectrometer of i-Red Infrared Systems with a probe in transmission mode was used. The spectrometer operates at a spectral range of 12,000–3800 cm^−1^ (833–2632 nm) and with a resolution of 1.5 cm^−1^. The probe was connected to the spectrometer using fiber optics and the spectral data was collected with near infrared process spectrometer software (NIPS). The chemometric evaluation of the measured spectra was carried out using the Thermo GRAMS/AI software package from Thermo Fisher Scientific. 

For a single spectrum, 50 scans (10 scans per second) were averaged. For each setting, 5 spectra were used to create a chemometrical model. To avoid drift effects caused by environmental or other parasitic effects, the measurement settings were chosen randomly.

### 2.8. Evaluation of NIR Data

The key element of chemometric modelling is to find relations between the composition of the sample, particle size, and/or mechanical properties. This procedure is extensive, due to the fact that NIR detects combinations of vibrations and overlapping bands. Therefore, a statistic calculation of a chemometric model is necessary to find relations between spectral data and properties. The difficulty in building a chemometric model is the problem of finding the appropriate algorithm, preprocessing method, and the wavelength range in which the desired property is imaged significantly. Therefore, NIR measurements require reference investigations to achieve this link between mechanical properties and spectral data. It is of immense importance for the accuracy of the chemometric model that these values be as precise as possible [19,20].

The multivariate data applied by NIR spectra are multi-dimensional (n-dimensional space) and it is therefore necessary to project the data on a two dimensional plane. This procedure is defined mathematically as an eigenvalue problem [23]:

(1) Z·e=e·λ

where *Z*: a square matrix (composed of NIR spectral data); *e*: eigenvector; λ: eigenvalue (composed of the reference values).

All performable evaluation methods, such as principal component analysis (PCA), principal component regression (PCR), or partial least squares (PLS 1 and PLS 2) basically work with this approach. It is advantageous to exclude some regions with irrelevant spectral information. This can be done by calculating an absorption spectra from two different spectra. This absorption spectrum shows those wavelength regions with the highest difference and preferably low signal noise. This region can then be chosen to achieve highly correlating chemometrical models [23].

Pre-processing is often beneficial in getting rid of parasitic effects, such as light straying caused by irregularities in the specimen, which lead to different path lengths when the light passes through the sample. A way to achieve a correction of these varying path lengths is to normalize the spectra to correct simple nonlinearities or to use algorithms, such as standard normal variate transformation (SNV) or multiplicative scatter correction (MSC) [23].

**Figure 1:**
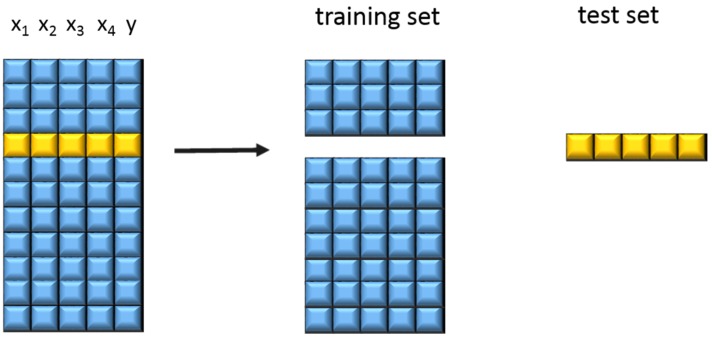
Principle of cross validation (according to Lohninger [23]).

The different samples were measured, pre-treated, and then the PLS 1 algorithm was used to generate a linear calibration model for calculating the responses from the measured NIR data using reference values. To gain information about the performance of a model, a cross validation was performed. Cross validation provides the probability to estimate how accurately a predictive model will perform in practice. One round of cross-validation involves partitioning a sample of data into complementary subsets, performing the analysis on one subset (called the training set, which is usually the larger one), and validating the analysis on the other subset (called the testing set). To reduce variability, multiple rounds of cross-validation are performed using different partitions, and the validation results are averaged over the rounds. The principle of cross validation can be seen in Figure 1.

The quality and the predictive ability of the model is rated basically with the coefficient of determination *R*^2^ and the root mean square error of cross validation (RMSECV). *R*^2^ (values between 0 and 100%) shows the correlation of the NIR data with the reference values of the response parameter. The coefficient of determination *R*^2^ should lie above 90% for quantitative calculation. Additionally, a precise model would have a RMSECV as low as possible. The number of factors used is determined by the number of process parameters (e.g., temperature, mixing speed, *etc.*) having an impact on the investigated property. Nevertheless, too many factors may lead to chemometric models that are less stable [24].

RMSECV and *R*^2^ are calculated as follows:

(2) $$R^{2}=1-\frac{\sum_{i=1}^{n}\left(Y_{pi}-{\bar{Y}}_{j}\right)}{\sum_{i=1}^{n}\left(Y_{ai}-{\overset{=}{Y}}_{j}\right)};\;\text{RMSECV}=\sqrt{\frac{\sum_{i=1}^{n}\left(Y_{ai}-Y_{pi}\right)}{n}}$$

Where *Y*_a_ is the actual measurement value; *Y*_p_ is the predicted value; $\bar{Y}$, $\overset{=}{Y}$ are the mean values.

The predicted values are then calculated according to the eigenvalue problem explained further.

(3) $$Z\cdot e=e\cdot Y_{p}$$

## 3. Results and Discussion 

Generally, a run was neglected for a calculation if it significantly decreased the quality of the chemometric model. Five of the 23 runs presented in this paper were neglected—this is statistically acceptable. A neglect from single runs is sometime necessary due to the fact that some material properties, e.g., *G*′, are very sensitive on variations in process parameters, e.g., temperature, sample taking, and point-of-measuring, leading to large deviations in single measured values. Keeping such “inaccurate runs” can lead to non-correlating spectral data and consequently to less accurate chemometric models. 

### 3.1. Correlation of NIR Spectroscopy with Tensile Strength [24]

**Figure 2:**
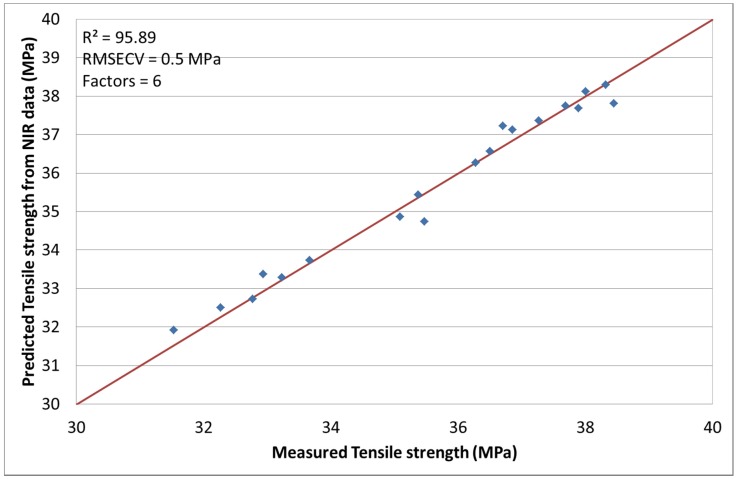
Predicted tensile strength values by NIR *versus* measured.

A very good correlation of the tensile strength and NIR spectra was achieved by designing an optimized chemometric model. The chosen spectral pre-treatment methods were mean center and SNV. This model gives an excellent coefficient of determination *R*^2^ = 95.89% with a RMSECV of 0.5 MPa and a factor number of 6. The NIR spectral data and the tensile strength for run 3 did not correlate well and, thus, the model was calculated without this data to improve the quality of the chemometric model. Figure 2 shows the results for the predicted tensile strength from NIR data *versus* the measured values.

### 3.2. NIR Spectroscopy with Young’s Modulus [24]

**Figure 3:**
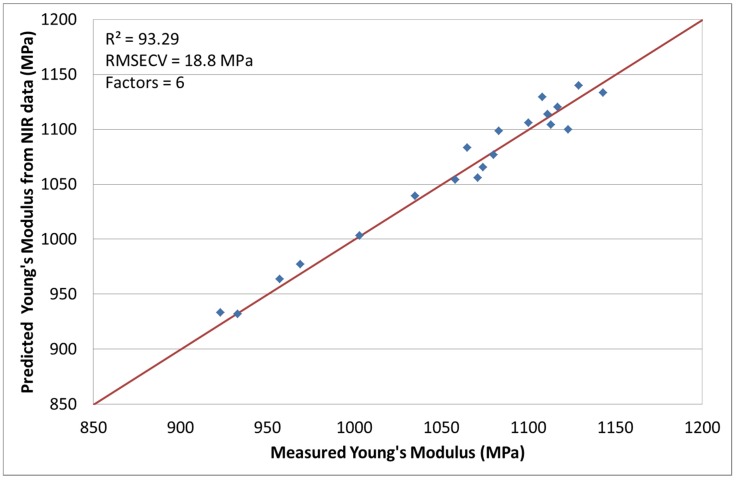
Predicted Young’s modulus values by NIR *versus* measured.

The second response of interest is the Young’s modulus. A very good correlation was obtained by optimizing the chemometric model with mean centering and normalization. The coefficient of determination, calculated with a factor number of 6, was *R*^2^ = 93.29% with a RMSECV of 18.8 MPa. Figure 3 shows the measured Young’s modulus *versus* the predicted values from the chemometric model. No run had to be excluded for this calculation.

### 3.3. NIR Spectroscopy with D-Spacing

**Figure 4:**
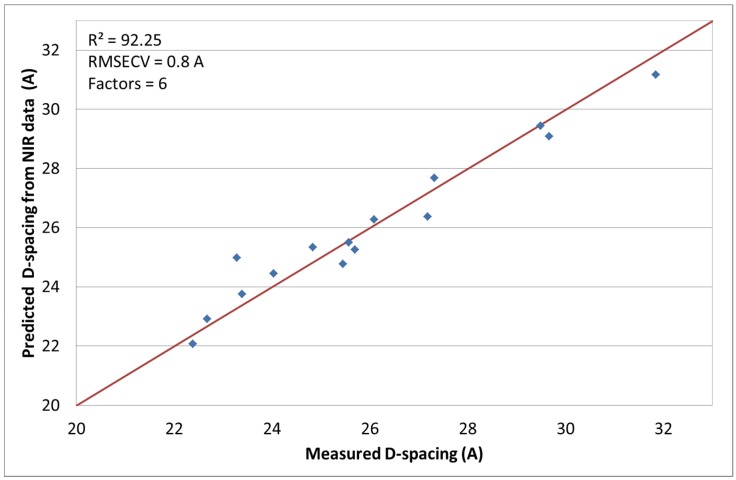
Predicted D-spacing values by NIR *versus* measured.

The third response of interest is the D-spacing obtained by XRD measurements. This measurement is useful for characterizing nanocomposites and is related to the distance between layered silicate tactoids. The chemometric model was optimized with mean centering and multiplicative scatter correction (MSC) [24]. The coefficient of determination obtained was *R*^2^ = 92.25% (factors = 6) with a RMSECV of 0.8 A. Figure 4 shows the results of the measured and calculated values. Runs 1, 10, 15 did not correlate well and were, thus, not included in this set. The reasons for this has been explained earlier.

### 3.4. NIR Spectroscopy with Interparticle Distance per vol. % Clay [24]

**Figure 5:**
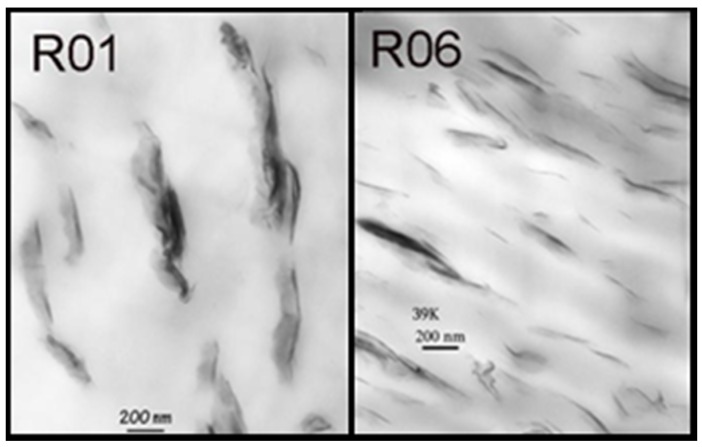
TEM-images for Run 1 (R01) and Run 6 (R06) [21].

Another response of interest was the interparticle distance per vol. % clay obtained from TEM data. An example for the investigated nanocomposites is shown in Figure 5.

**Figure 6:**
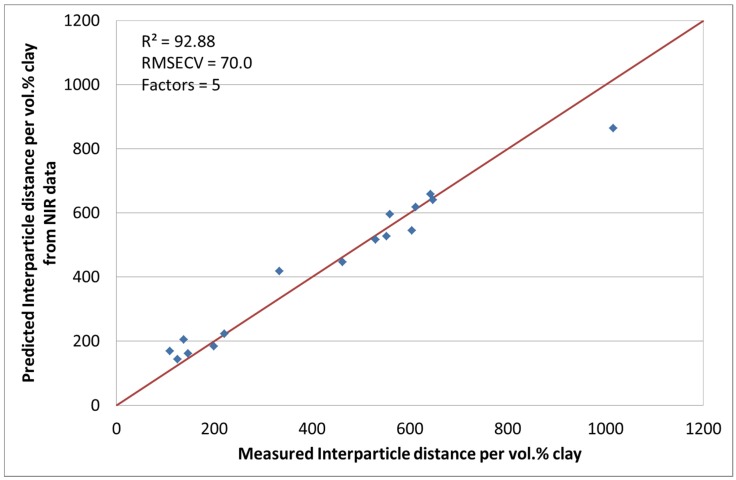
Predicted interparticle distance per vol. % clay values by NIR *versus* measured.

Although this measurement is the standard investigation method for the characterization of the interparticle distance of the silicate layers, it is time consuming and expensive. The chosen spectral pre-treatment methods were mean centering and MSC. With the use of five factors, the coefficient of determination (*R*^2^) was calculated to be 92.88% with a RMSECV of 70. Figure 6 shows the actual and calculated data. During the chemometric modelling run, 3 and 10 were excluded. The reasons for this have been explained earlier.

### 3.5. NIR Spectroscopy with G′ at 0.01 Hz 

**Figure 7:**
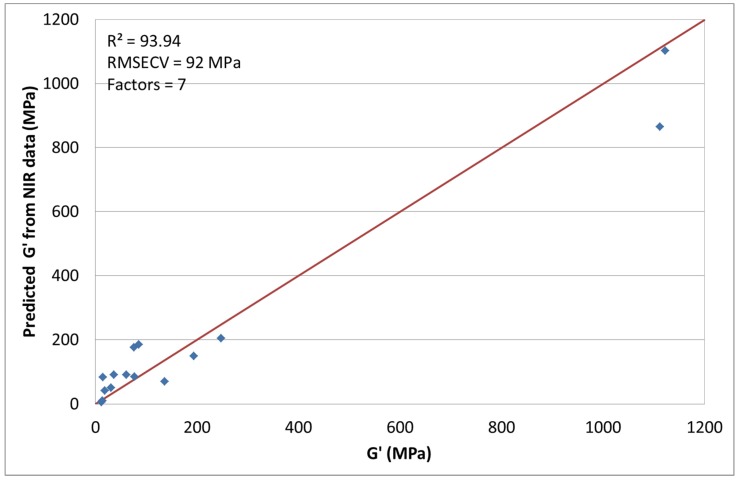
Predicted G’ by NIR *versus* measured.

We also decided to correlate the rheological measurements with the near infrared spectroscopic data. *G*′, at a frequency of 0.01 s^−1^, was correlated and the spectral pretreatment method chosen was mean centering. With the use of seven factors, the coefficient of determination was calculated as *R*^2^ = 93.94% with a RMSECV of 92 MPa (see Figure 7). During the chemometric modelling runs 7, 8, 19, 20, and 21 had to be excluded. The correlation values seem at first sight quite usable, however the high RMSECV shows the high variation of the model. The good value for the coefficient of determination results primarily from the data points at about 1200 MPa. This phenomenon is the so-called leverage effect. Through the two values that are very far away from the rest of the data points, an apparent linearity is simulated, which actually does not exist. If these two data points are excluded, no correlation can be calculated. This example also shows that the coefficient of determination should not be used alone [20]. A possible reason for the non-correlation with the recorded spectral data could be the temperature and aggregate state dependency. *G*′ is the dominant part in the solid phase of the polymer. It decreases considerably with increasing temperature. Since spectral data and the storage modulus were measured at different temperatures and in different aggregate states, a correlation can only be found if all investigated composites are changing by the same factor. If this is not completely the case, no or only bad correlations can be found.

### 3.6. NIR Spectroscopy with G″ at 0.01 Hz [24]

**Figure 8:**
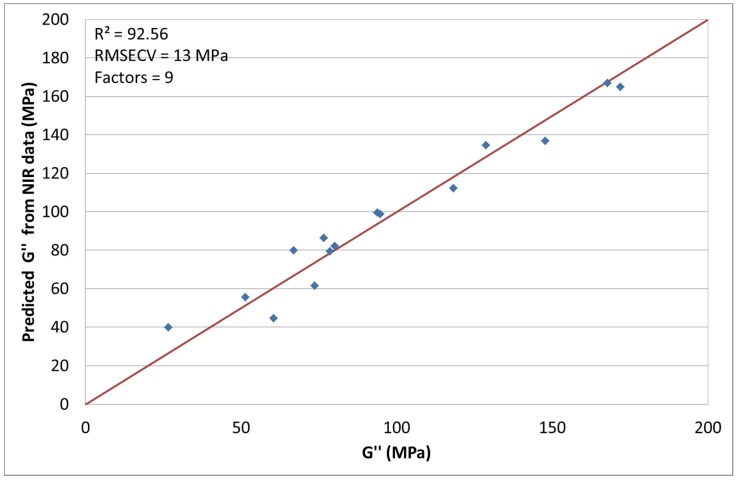
Predicted *G*″ by NIR *versus* measured.

The other rheological response of interest was G″ at a frequency of 0.01 s^−1^. The chosen spectral pretreatment methods were mean centering and MSC. With the use of 9 factors *R*^2^ was calculated to be 92.56% with a RMSECV of 13 MPa (see also Figure 8). During this chemometric modelling runs 4, 6, 11, 19 and 20 had to be excluded like mentioned before due to small variations during processing and measuring. One possible cause, as already mentioned at the discussion of the storage modulus, could be the temperature and the aggregate state dependency. The loss modulus is the dominant part in the molten phase of the polymer. Although there is a temperature dependency, it is not as distinct compared to the storage modulus. Even though the spectral data and the loss modulus were measured at different temperatures and in different states, the correlation is better here due to the lower temperature and aggregate state dependency. By removing the above-mentioned single runs, a useful model can be calculated.

## 4. Conclusions

This work shows that the tensile strength, the Young’s modulus, the D-spacing and the interparticle distance per vol. % clay exhibit good correlations with predictions from near infrared spectroscopy measurements. With some restrictions, due to the fact that rheological measurements are very sensitive to process history, it was even possible to correlate the rheological data *G*″. A correlation for *G*′ could not be realized due to the stronger temperature and aggregate state dependency compared to *G*″.

Very promising chemometric models have been obtained. Although the properties and the spectra were not collected from the same physical samples, they were obtained on samples from the same processing runs. The fact that XRD and TEM measurements are expensive and time consuming, the possibility of characterizing samples by NIR is the most important insight of these investigations. Although the correlation with the rheological data required an exclusion of more data points and an increase in the factor number, it could be shown that the NIR technology is able to indicate rheological changes in polymer nanocomposites. Because of the certainty that the factor number is more or less reflecting the number of external coefficients, such as temperature or pressure having an impact on the investigated properties, it can be seen that rheological data are more prone to external influences compared to the other investigated properties. 

Since near infrared measurements can be made in-line, directly in the melt, real time characterization and advanced quality control during production of nanocomposites could be achieved. This would lead to faster development of new nanocomposites and their components, whereas the processing of the composites would be optimised with reduced costs. However, implementing such a system would need to be approached with care, to ensure accurate assessments are being made. In practice it would be beneficial if all the spectral data where measured directly in the melt during the production of the nanocomposites. The DoE should cover the area of material property, which is of major interest regarding quality control. Nevertheless, it must be mentioned that the accuracy of a calculated chemometric model can, at most, be as good as the reference values.
